# CD301 and LSECtin glycan-binding receptors of innate immune cells serve as prognostic markers and potential predictors of immune response in breast cancer subtypes

**DOI:** 10.1093/glycob/cwae003

**Published:** 2024-01-11

**Authors:** Anne-Sophie Wegscheider, Irina Wojahn, Pablo Gottheil, Michael Spohn, Joseph Alfons Käs, Olga Rosin, Bernhard Ulm, Peter Nollau, Christoph Wagener, Axel Niendorf, Gerrit Wolters-Eisfeld

**Affiliations:** MVZ Prof. Dr. med. A. Niendorf Pathologie Hamburg-West GmbH, Institut für Histologie, Zytologie und Molekulare Diagnostik, Lornsenstr. 4, 22767 Hamburg, Germany; MVZ Prof. Dr. med. A. Niendorf Pathologie Hamburg-West GmbH, Institut für Histologie, Zytologie und Molekulare Diagnostik, Lornsenstr. 4, 22767 Hamburg, Germany; Peter Debye Institute for Soft Matter Physics, Leipzig University, Linnéstr. 5, 04103 Leipzig, Germany; Department of Pediatric Hematology and Oncology, University Medical Center Hamburg-Eppendorf, Martinistr. 52, 20246 Hamburg, Germany; Research Institute Children's Cancer Center, Martinistr. 52, 20246 Hamburg, Germany; Bioinformatics Core, University Medical Center Hamburg-Eppendorf, Martinistr. 52, 20246 Hamburg, Germany; Peter Debye Institute for Soft Matter Physics, Leipzig University, Linnéstr. 5, 04103 Leipzig, Germany; MVZ Prof. Dr. med. A. Niendorf Pathologie Hamburg-West GmbH, Institut für Histologie, Zytologie und Molekulare Diagnostik, Lornsenstr. 4, 22767 Hamburg, Germany; Unabhängige Statistische Beratung Bernhard Ulm, Kochelseestr. 11, 81371 München, Germany; Department of Pediatric Hematology and Oncology, University Medical Center Hamburg-Eppendorf, Martinistr. 52, 20246 Hamburg, Germany; Research Institute Children's Cancer Center, Martinistr. 52, 20246 Hamburg, Germany; Medical Faculty, Universität Hamburg, Martinistr. 52, 20246 Hamburg, Germany; MVZ Prof. Dr. med. A. Niendorf Pathologie Hamburg-West GmbH, Institut für Histologie, Zytologie und Molekulare Diagnostik, Lornsenstr. 4, 22767 Hamburg, Germany; Department of General, Visceral and Thoracic Surgery, University Medical Center Hamburg-Eppendorf, Martinistr. 52, 20246 Hamburg, Germany

**Keywords:** breast cancer, C-type lectins, glycan-binding receptors, immune cells, protein domain histochemistry

## Abstract

Glycosylation is a prominent posttranslational modification, and alterations in glycosylation are a hallmark of cancer. Glycan-binding receptors, primarily expressed on immune cells, play a central role in glycan recognition and immune response. Here, we used the recombinant C-type glycan-binding receptors CD301, Langerin, SRCL, LSECtin, and DC-SIGNR to recognize their ligands on tissue microarrays (TMA) of a large cohort (*n* = 1859) of invasive breast cancer of different histopathological types to systematically determine the relevance of altered glycosylation in breast cancer. Staining frequencies of cancer cells were quantified in an unbiased manner by a computer-based algorithm. CD301 showed the highest overall staining frequency (40%), followed by LSECtin (16%), Langerin (4%) and DC-SIGNR (0.5%). By Kaplan-Meier analyses, we identified LSECtin and CD301 as prognostic markers in different breast cancer subtypes. Positivity for LSECtin was associated with inferior disease-free survival in all cases, particularly in estrogen receptor positive (ER+) breast cancer of higher histological grade. In triple negative breast cancer, positivity for CD301 correlated with a worse prognosis. Based on public RNA single-cell sequencing data of human breast cancer infiltrating immune cells, we found *CLEC10A* (CD301) and *CLEC4G* (LSECtin) exclusively expressed in distinct subpopulations, particularly in dendritic cells and macrophages, indicating that specific changes in glycosylation may play a significant role in breast cancer immune response and progression.

## Introduction

Breast cancer emerged as the most frequently diagnosed cancer worldwide in 2020, registering an alarming 2.26 million new cases and causing nearly 685,000 breast cancer-related fatalities, according to data from the International Agency for Research on Cancer and the World Health Organization (IARC/WHO 2023).

Traditional clinical and pathological indicators, such as tumor size, grade, and lymph node involvement, along with the evaluation of specific biomarkers like estrogen receptor (ER), progesterone receptor (PR), and human epidermal growth factor receptor 2 (HER2), have historically played crucial roles in the staging and prognosis of breast cancer (Rakha et al. 2010; Gao and Swain 2018). In addition, over the last two decades, there has been a significant increase in research focused on genetic markers and the application of multigene assays for the purpose of classifying prognosis and forecasting treatment outcomes in breast cancer (Tschodu et al. 2022).

However, as breast cancer is a heterogeneous disease, it is still difficult to give valid prognostic information in many cases, e.g. concerning special histological types or triple negative breast cancers, illustrating the need for further tools and markers (Weigelt et al. 2010; Wegscheider et al. 2021; Derakhshan and Reis-Filho 2022).

Besides DNA, RNA, proteins and metabolites, glycans are considered as biomarkers in breast cancer. Glycans form the glycocalyx, a dense layer of carbohydrates on the surface of eukaryotic cells (Gagneux et al. 2022). Because of their pronounced structural diversity, glycans are of great interest with respect to informational content, particularly in cancer. With the identification of glycan-binding receptors, a fundamental change has taken place in glycobiology in recent years (Varki 2017). In addition to the established extrinsic recognition of glycans by microorganisms, processes of intrinsic recognition were identified in different functional contexts. With regard to cancer, cell adhesion and immune recognition should be mentioned in particular (Taylor and Drickamer 2007; Pinho et al. 2023).

In general, glycan-binding receptors interact with the sugar moieties via carbohydrate recognition domains (CRDs). Based on structural features of the CRDs, different groups of glycan-binding receptors can be distinguished. Among the largest groups are the C-type lectins, in which sugars are bound to the CRD in the presence of bivalent cations (Taylor and Drickamer 2019). It has been suggested that glycan-binding receptors of the C-type lectin family recognize glyco-codes as novel immune checkpoints (RodrIguez et al. 2018).

When glycan-binding receptors are employed as diagnostic tools, the sugars they adhere to hold functional significance, setting them apart from plant lectins and monoclonal antibodies. Here, we used CD301, Langerin, SRCL, LSECtin, and DC-SIGNR human C-type lectins, each known for their distinct glycan binding specificities. CD301 predominantly recognizes truncated O-GalNAc glycans such as the Tn and STn antigens, as well as Neu5Gc (Mortezai et al. 2013; Nollau et al. 2013). Langerin exhibits affinity for blood group antigens A and B (Feinberg et al. 2011), as well as sulfated and mannosylated glycans, keratan sulfate (Tateno et al. 2010) and beta-glucans (de Jong et al. 2010). SRCL demonstrates binding capability to Lewis structures X and A (Blanas et al. 2018) as well as various carbohydrates, including Gal-type ligands, D-galactose, L- and D-fucose, GalNAc, and T/Tn antigens, in a calcium-dependent manner (Yoshida et al. 2003). LSECtin exhibits high selectivity for glycoproteins terminating in GlcNAcβ1-2Man (Powlesland et al. 2008) and recognizes a pair of positional glycan isomers (LDN3 and LDN6), along with a nonelongated GlcNAc4Man3 N-glycan (Bertuzzi et al. 2022). DC-SIGNR specifically binds to high mannose glycans (Feinberg et al. 2007).

In this study, we used probes of glycan-binding receptors to detect their ligands on breast cancer tissues from 1,859 patients, each with a clinical follow-up period of at least ten years. Our findings indicate that glycoreceptor ligands are particularly prevalent in specific tumor entities, suggesting their potential as diagnostic and prognostic tools, as well as therapeutic targets. Notably, CD301 ligands emerge as prognostic markers in triple-negative breast cancer, and LSECtin ligands show promise in tumors that pose challenges in prediction and therapeutic decision-making (Mitra and Dey 2015).

## Results

### In vitro validation of glycan-binding receptors specificities

**Fig. 1:**
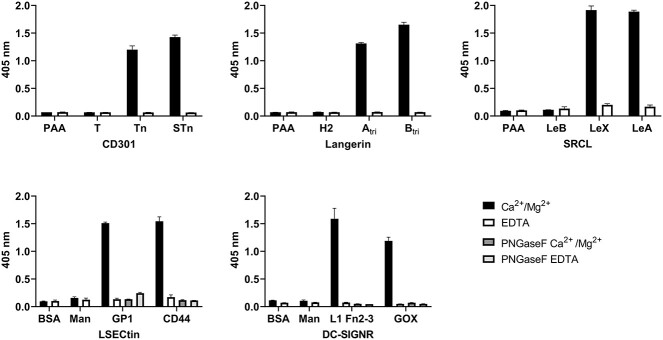
In vitro validation of glycan-binding receptor specificities. GBR binding was assessed by ELISA using glycoconjugates and defined glycoproteins as positive and negative controls in the presence and absence of Ca^2+^/Mg^2+^. CD301 specifically binds to Tn and STn antigens but not to T antigen (core 1) glycoconjugates or the polyacrylamide (PAA) backbone. SRCL exhibits specific binding to glycoconjugates Lewis structures a and X, but not to B. Langerin selectively binds to glycoconjugates of ABO blood groups a and B, but not to the H structure. LSECtin binds recombinant Ebola virus glycoprotein 1 and human CD44 and DC-SIGNR binds L1CAM Fn2-3 produced in HEK293S GnTI- cells and glucose oxidase (GOX_ASPGN) from *Aspergillus niger* in the presence of Ca^2+^/Mg^2+^. PNGase F digest resulted in a loss of GBR binding.

Prior to utilizing the recombinant glycan-binding receptors (GBRs) CD301, Langerin, SRCL, LSECtin, and DC-SIGNR for protein domain histochemistry on tissue microarrays, we thoroughly assessed their functionality and glycospecificity. To accomplish this, we conducted ELISA-based binding assays using immobilized glycoconjugates or defined glycoproteins as positive and negative controls. As bivalent cations are essential for binding of C-type family glycan-binding receptors, we conducted binding studies simultaneously, with one set in the presence of Ca^2+^ and Mg^2+^ ions, and the other set with EDTA included in the binding reaction to serve as an additional negative control (Fig. 1). Our results demonstrate that in the presence of bivalent cations, CD301 binds specifically to Tn and STn antigens but not T antigen (core 1) glycoconjugates or the polyacrylamide (PAA) backbone. SRCL exhibits specific binding to glycoconjugates carrying Lewis structures A and X, but not to Lewis B structures. Langerin selectively binds to glycoconjugates of ABO blood groups A and B, but not to the H structure. As no glycoconjugates were available for LSECtin and DC-SIGNR, we used purified glycoproteins with documented specificity for each respective glycan-binding receptor (Powlesland et al. 2008; Tang et al. 2010). We also included PNGase F digests as an additional control to confirm the binding of the glycan-binding receptors to N-glycosidic glycan structures. Our binding studies reveal that in the presence of Ca^2+^/Mg^2+^, LSECtin binds specifically to recombinant human CD44 and to glycoprotein 1 (GP1) from Ebola virus, both produced in HEK293 cells. We also confirm the binding and specificity of DC-SIGNR using recombinant L1CAM Fn2-3 (fibronectin type-III) expressed in HEK293S GnTI- cells and recombinant glucose oxidase produced in *Aspergillus niger* (Takegawa et al. 1991). Treatment of these proteins with PNGase F prior to the binding reaction results in a complete loss of GBR binding. In addition, we observe no binding in the presence of EDTA. To validate the assay, negative control spots in the glycan ELISA were effectively probed using biotinylated plant lectins with well-defined glycan specificities (Fig. S1A). Further, dilution curves of glycan-binding receptor complexes illustrate asymptotic dose-response curves, thereby indicating equilibrium conditions (Fig. S1B). Taken together, our in vitro binding studies demonstrate that all five glycan-binding receptors are functionally active and bind specifically to defined glycan structures in a Ca^2+^/Mg^2+^- and glycan dependent manner.

### Validation of glycan-binding receptor staining

For optimization and validation of our staining procedure, we used pancreatic tissue from conditional *C1galt1c1* knockout mice, specifically expressing the glycan structures recognized by CD301 in contrast to control mice (Wolters-Eisfeld et al. 2018). Staining of formalin fixed, paraffin embedded (FFPE) tissue sections was performed in parallel in the presence of Ca^2+^/Mg^2+^ or EDTA serving as a negative control. For CD301, we observed a highly specific binding to the apical epithelial lining of the pancreas in *C1galt1c1* knockout mice while pancreatic tissue of the wild type mice was negative (Fig. S2A). To further validate the binding using CD301, Langerin, SRCL, LSECtin, and DC-SIGNR, respectively, on tissue microarrays (TMAs) we used tissue samples of normal human and cancerous origin including colon, endometrium, liver, lung, ovary, pancreas and nervous tissue (Table S1, Fig. S2B). Compared to the EDTA controls, we observed strong and specific staining of CD301, Langerin, SRCL, DC-SIGNR and LSECtin limited to the apical surface of normal and/or cancerous cells; staining of the stroma was negative. For unknown reasons, we observed substantial background binding of SRCL with high frequency in a variety of normal and cancerous tissues, preferentially in the nucleus of epithelial and stromal cells (Fig. S3). In summary, based on our test staining we observed highly specific binding of CD301, Langerin, LSECtin and DC-SIGNR on FFPE tissue sections of mouse and/or human origin. Due to substantial and non-controllable background staining of SRCL, we excluded SRCL from further analysis.

### Staining of TMAs by glycan-binding receptors

**Fig. 2:**
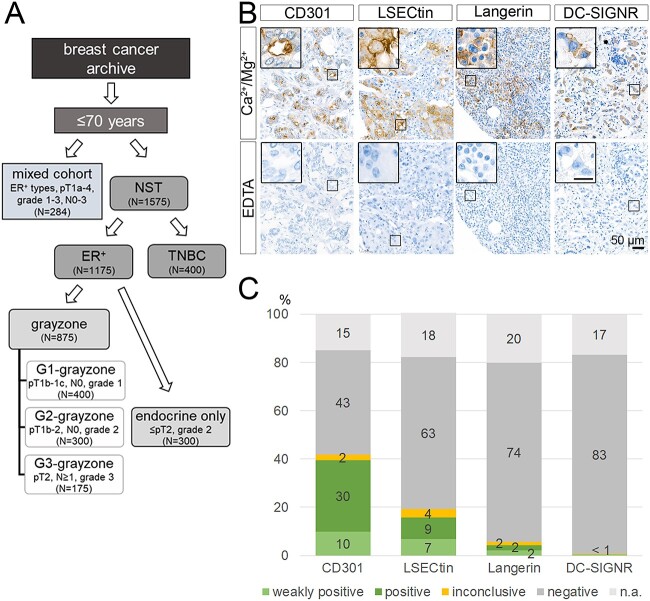
Differential glycan profiles in breast cancer subtypes. A) an illustrative summary depicting the various cohorts of breast cancer samples and their respective subtypes. B) Representative tissue sections of breast cancer specimens stained with CD301, LSECtin, Langerin and DC-SIGNR, respectively. Binding reactions were performed in the presence of bivalent cations Ca^2+^/Mg^2+^. Parallel incubation in the presence of EDTA served as negative control. The scale bar in the inserts corresponds to 20 μm. C) Percental staining frequencies of CD301, LSECtin, Langerin and DC-SIGNR staining in the entire cohort of breast cancer samples (*n* = 1859).

To address the important clinical question whether glycan signatures are of diagnostic and prognostic value in breast cancer, we analyzed a representative cohort of *n* = 1,859 invasive breast cancer samples from our tumor archive. We excluded patients aged older 70 years to eliminate cases with potential non-tumor-related death. Subsequently, we divided our cohort into two primary groups: the first group consisted of ER-positive tumors (*n* = 1,175), while the second group comprised triple-negative breast cancers (*n* = 400), characterized by the absence of ER, PR, and HER2/neu receptor expression. In order to eliminate potential confounding variables and provide prognostic insights into intermediate-risk groups, currently challenging to predict (Phung et al. 2019; Wegscheider et al. 2021) we conducted a more nuanced categorization subdividing the ER-positive cases in four distinct subgroups, which we refer to as G1-, G2-, and G3-grayzones and the endocrine only subgroup. These subcategories are defined based on patients who exhibited variations in their pathological tumor stage and grade and underwent endocrine therapy or endocrine therapy, combinational chemotherapy or both after surgery (Fig. 2A).

After sampling and fabricating of TMAs, we stained a total number of 21 TMAs comprising 1,859 breast cancer samples with the glycan-binding receptors CD301, Langerin, LSECtin and DC-SIGNR, respectively (Fig. 2B). As negative control, we performed staining reactions in parallel for each TMA in the presence of EDTA. Evaluated by microscopic examination, CD301 showed the most frequent positive staining in 40% of all cases, followed by LSECtin with 16% and Langerin with a positivity rate of 4% (Fig. 2C), respectively, while positivity for DC-SIGNR was observed in only 0.5% of the analyzed cases.

**Fig. 3:**
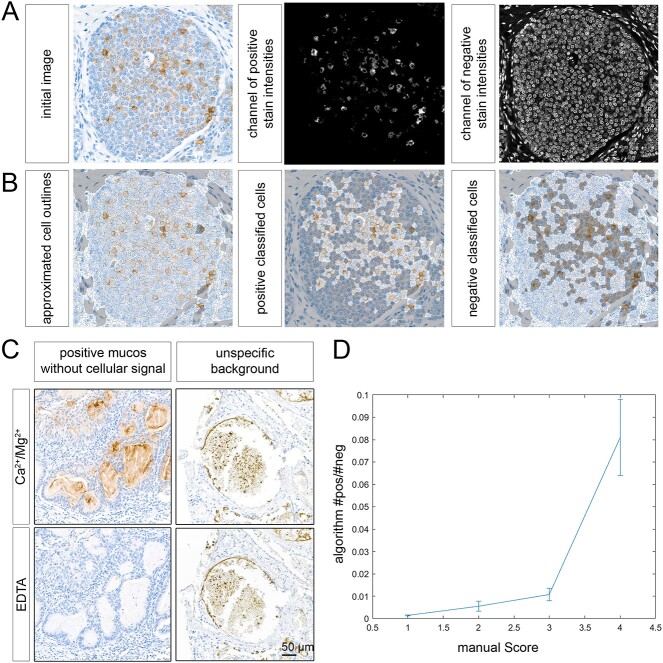
Consecutive steps and validation of the algorithm-based evaluation of breast cancer TMAs. A) Color deconvolution and transformation of a CD301-stained, representative tissue section into positive and negative signals representing GBR binding and nuclear counter staining. B) Determination of cellular outlines and separation of positive and negative stained cells. C) Example of GBR binding signals (here: CD301 positive mucus, while cancer cells are negative) and unspecific background which might produce false positives in an IT based evaluation D) comparison of manual and algorithm-based evaluation. The medians of the algorithmic score are shown for each respective manual score. Confidence intervals are calculated using bootstrap resampling with 10,000 resamplings. Harrels concordance index between manual and algorithmic score equals 0.66.

Given the large number of samples and limitations of potentially biased, microscopic evaluation by individual investigators, we developed and applied a computer-based algorithm to determine staining frequencies of breast cancer cells in a quantitative, unbiased and fully automated manner. For this purpose, we digitized microscopic slides at 40-fold magnification using a 3D HisTech high-throughput slide scanner. By color deconvolution, we transformed the RGB intensity space into a two-color space representing the nuclear (hematoxylin, blue) and GBR stain (chromogen DAB, brown), respectively (Fig. 3A). Using the nuclei as initialization points, we approximated the cellular outlines halfway between neighboring nuclei, classified positive and negative stained cells by their signal intensities and counted the positively stained cells (Fig. 3B).

During microscopic examinations, we observed tissue sections with pronounced background staining, and in certain samples, intraluminal mucus exhibited positive staining while cancer cells did not, as illustrated in Fig. 3C. Despite the challenges posed by such staining patterns, our algorithm adeptly identified stained mucinous regions as false positives, discerning the absence of cell nuclei and effectively excluding these areas from consideration in individual TMA specimens.

While manual evaluation allows for the discrimination of positively stained cells and nonspecific background, particularly with the use of EDTA negative controls, our investigation delves into whether these signals could potentially compromise the digital assessment. To assess the consistency and robustness of our algorithm further, we conducted a comparative analysis between the results generated by our algorithm and those obtained through microscopic evaluation, categorizing findings as negative, inconclusive, scattered positive, and positive (see Fig. 3D). The results unveiled a high degree of concordance between the algorithm-based analysis and microscopic assessment, affirming the reliability of our scoring algorithm, which includes the accurate evaluation of challenging staining patterns.

### LSECtin and CD301 are prognostic markers in different breast cancer subtypes

**Fig. 4:**
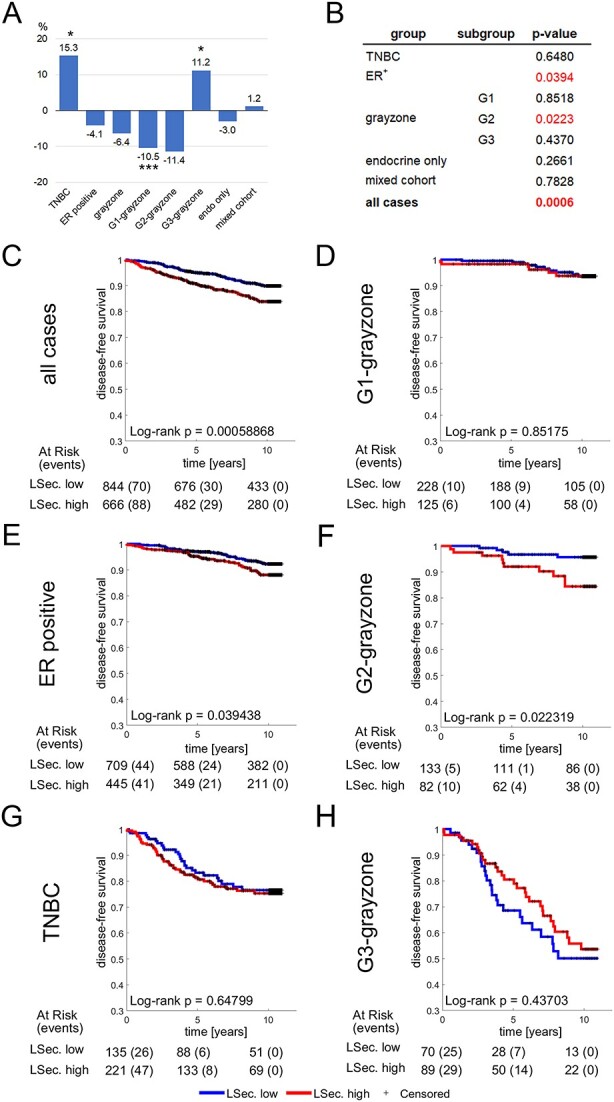
LSECtin is a potential prognostic and diagnostic marker of breast cancer. A) Frequencies of LSECtin staining in different breast cancer subgroups in comparison with the average staining frequencies. B) Summary of Kaplan-Meier log-rank p-values for the different breast cancer subgroups. Significant associations between staining frequencies and disease-free survival are displayed in red. C–H) Kaplan-Meier plots for various breast cancer subgroups showing either a significant deviation of the mean frequency or a significant effect on disease-free survival.

After validating our scoring algorithm, we proceeded to assess the staining frequencies of the glycan-binding receptors LSECtin, CD301, and Langerin, respectively. Due to the limited number of positive cases (*n* = 9), DC-SIGNR was not further analyzed. In the case of LSECtin, we observed less frequent staining in G1 and G2 gray zone tumors and more frequent staining in G3 gray zone tumors and TNBC, as compared to the average positive staining frequency in our cohort of breast cancers (Fig. 4A). When we conducted Kaplan-Meier analysis to correlate staining frequencies with disease-free survival, we identified a highly significant dependency between disease-free survival and positive staining frequencies for LSECtin across all breast cancer cases (Fig. 4B and C). Notably, we found that high staining frequencies of glycan structures recognized by LSECtin were associated with a worse prognosis, and this association was particularly pronounced in the subgroup of ER+ breast cancers, specifically in tumors of the G2 gray zone (Fig. 4E and F), with G3 gray zone tumors showing a trend (Fig. 4H). Conversely, we did not observe a significant association between disease-free survival and positive staining for LSECtin in G1 gray zone and TNBC (Fig. 4D and G).

**Fig. 5:**
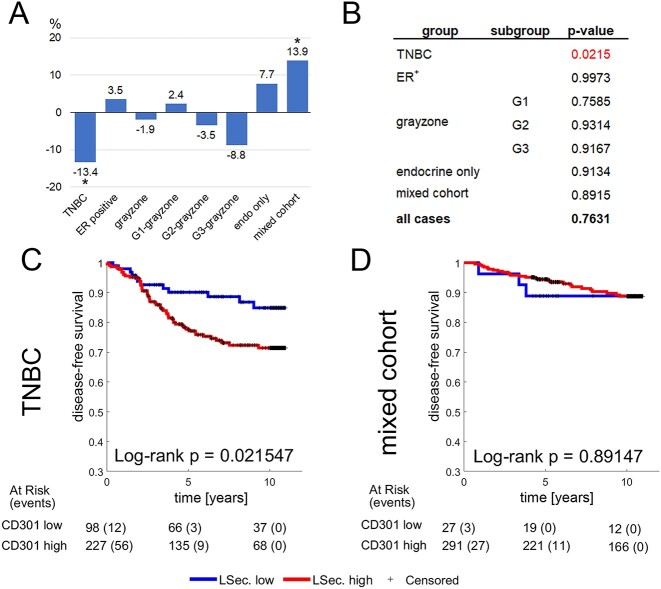
CD301 is a potential prognostic marker in TNBC. A) CD301 staining frequency in breast cancer subgroups compared to the average staining frequencies. B) Summary of log-rank p-values in breast cancer subgroups. Significant associations between staining frequencies and disease-free survival are shown in red. C and D) Kaplan-Meier plots for TNBC breast cancer subtype and mixed cohort.

Regarding CD301, we noted on average a lower staining frequency in TNBC and elevated levels in the mixed cohort (Fig. 5A). Kaplan-Meier analysis revealed a significant association between staining frequencies and disease-free survival in TNBC, where high staining frequencies of glycan structures recognized by CD301 were linked to a significantly worse prognosis (*p* = 0.0215) (Fig. 5B and C). We were unable to discern an effect on disease-free survival in the mixed cohort and ER+ breast cancers (Fig. 5D).

In the case of Langerin, we observed significantly less frequent staining in G2 gray zone tumors within our analyzed breast cancer samples compared to the average frequency of positive staining (Fig. S4A). Nevertheless, when we explored the correlation between staining frequencies and disease-free survival using Kaplan-Meier analysis, we found no significant association between disease-free survival and positive staining frequency for Langerin. This lack of correlation was consistent across all breast cancer cases and within specific subgroups (Fig. S4B).

In summary, our analysis of a large cohort encompassing various breast cancer subtypes and glycan-binding receptors revealed significant associations between disease-free survival and staining positivity in distinct subtypes of breast cancer. Notably, such associations were observed in TNBC with CD301 and in ER-positive breast cancer cases, specifically in non-special type (NST) tumors of intermediate grade (without lymph node metastases, tumor size between 0.5 cm and 2 cm) with LSECtin.

**Fig. 6:**
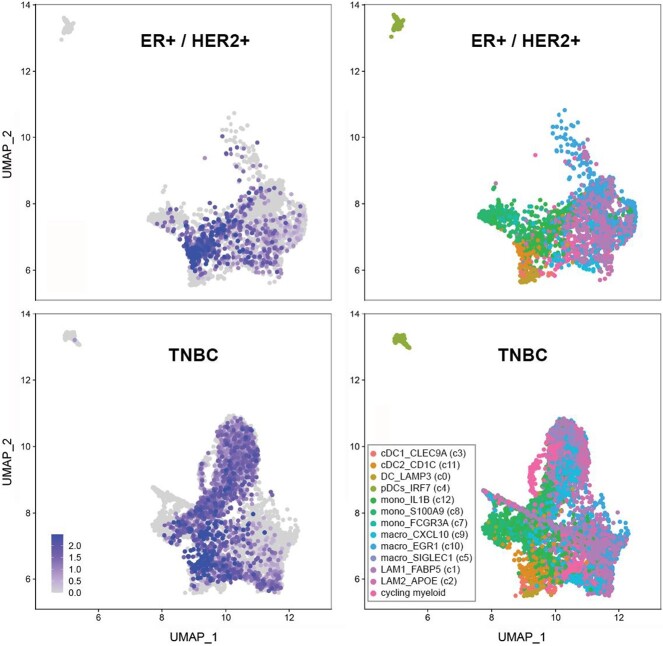
UMAP visualization of *CLEC10A* expression in the myeloid cell cluster of TNBC and HER2+/ER+ breast cancer cells. Relative levels of expression are given in the left panel; the corresponding cell subtypes of the myeloid cluster are shown in the right panel. UMAPs were generated from published single cell sequencing data (Wu et al.). Note the significant increase of LAM2_APOE+, LAM1_FABP5+ and EGR1+ macrophages in TNBC compared to HER2+/ER+ breast cancer cells.

Given the importance of CD301 ligands in breast cancer prognosis, particularly in TNBC, we interrogated the expression of *CLEC10A* in breast cancer tissues based on a recently published, high-resolution transcriptional landscape obtained by single cell sequencing of different types of breast cancer (Wu et al. 2021). Among different cancer types (ER+, HER2+ and TNBC) and other tumor-associated cells, we almost exclusively observed *CLEC10A* expression in the myeloid cell cluster (Fig. S5), preferentially in cDC2 and IL1B+ monocytes of which more than 40% of cells expressed CD301 (Fig. S6). When we separately analyzed TNBC and ER+ / HER2+ cancer cells, we found no major differences in levels of *CLEC10A* expression (Fig. S6), but significant differences in the number of *CLEC10A* expressing cells (Fig. 6). Approximately 30% more cells of the macrophage subtypes positive for LAM2_APOE, LAM1_FABP5 and EGR1, respectively, expressed *CLEC10A* in TNBC in comparison with ER+ / HER2+ breast cancer (Chi-squared *p* < 0.001, Table S4). EGR1+ macrophages resemble the M2-like phenotype, while LAM1_FABP5 and LAM2_APOE are representing lipid-associated macrophages (LAM), a subtype distinct from the M1−/ M2-phenotype. According to Wu et al. (Wu et al. 2021), the LAM1_FAB5 signature correlates with a worse survival.

Comparable to *CLEC10A*, we also observed expression of *CLEC4G* exclusively in the myeloid cell cluster (Fig. S5), most frequently in EGR1+ and LAM2_APOE + macrophages as well as cycling myeloid cells and SIGLEC1 + macrophages (Fig. S7, Table S4). Expression levels were relatively low in the different myeloid subtypes and we found no significant differences of *CLEC4G* expression levels and frequency between cancer types (Fig. S6, Table S4). Taken together, our data suggest that an immune response triggered by the Tn antigen is very likely driven by the immune cell composition of the breast cancer microenviroment, particularly by specific subpopulations of macrophages, rather than by differences in levels of *CLEC10A* expression on immune cells.

## Discussion

The objective of this study was to assess the suitability of glycan-binding receptor ligands as potential biomarkers for breast cancer. To utilize glycan-binding receptors as diagnostic and prognostic tools, several conditions must be met: (I) Glycan-binding receptors must serve as effective probes, capable of specifically binding their ligands within tissue sections. (II) A sufficient quantity of tumor tissues must be available to permit statistically meaningful conclusions. (III) A quantitative method for assessing receptor binding should be available. (IV) The clinical progress of cancer patients over a crucial time span must be meticulously documented. These prerequisites were met in the context of this study. Our probes targeting glycan-binding receptors demonstrate suitability for the precise detection of glycan ligands. In addition to the CD301 probe, the characteristics of which have been previously described in various studies (Samsen et al. 2010; Mortezai et al. 2013; Nollau et al. 2013), we have also developed probes for glycan-binding receptors such as SRCL, LSECtin, Langerin, and DC-SIGNR. All of these probes exhibited the capability to specifically detect their respective ligands, as demonstrated through biochemical binding studies.

To examine a sufficiently large number of tissue samples with corresponding patient follow-ups, it was essential for the probes to be effective in relation to the pre-treated tissues. This prerequisite was met for the CD301, LSECtin, and Langerin probes. Given that the activity of C-type lectins relies on the presence of divalent cations, we assessed non-specific staining by introducing EDTA. Unfortunately, we observed non-specific staining of cell nuclei with SRCL, rendering the evaluation of this receptor’s staining unreliable. DC-SIGNR exhibited sporadic binding to only a few tissue sections, supporting the hypothesis that DC-SIGNR primarily functions as a pathogen receptor, binding to mannans found in yeast and other fungi (Taylor and Drickamer 2019).

In routine histological diagnostics, formalin-fixed, paraffin-embedded (FFPE) tissues are commonly employed. Leveraging FFPE tissues arranged on tissue microarrays, we were able to stain tumor sections from 1,859 breast cancer patients using glycan-binding receptor probes and draw conclusions regarding the patients’ prognoses. To date, this represents the largest cohort of breast cancer patients in which the relationship between the expression of glycan-binding receptor ligands and the clinical outcomes of patients has been investigated.

When using glycan-binding receptors to detect ligands in tissue sections, the task of achieving unbiased quantification becomes apparent. Traditionally, immunohistochemical staining is examined under a microscope by experts and assessed semi-quantitatively, utilizing a scale from “-” to “+++.” This method carries the potential for introducing subjective interpretations. In our present study, we adopted an algorithm to differentiate between areas that displayed positive and negative staining within the sections.

Through the identification of cell nuclei, we generated digital images that not only conveyed the intensity of the positive signal but also quantified the number of stained cells. In a series of experiments, we compared the outcomes derived from computer-assisted analysis with the microscopic evaluations of the sections, demonstrating a high level of congruence. In contrast to microscopic evaluation, digital image analysis provides the distinct advantage of yielding objective results that can be correlated with clinical outcomes. To summarize, while human bias is often inherent, an algorithmic approach guarantees objectivity in our assessments.

The diagnostic and prognostic significance of glycan-binding receptor ligands in tissue sections was evaluated in a total of 1,859 breast cancer patients. When considering the entire patient population, only LSECtin was of prognostic significance. From a clinical point of view, the expression of LSECtin ligands in tumors of the G2 gray zone is particularly relevant. These are tumors of intermediate differentiation in which conventional diagnostic parameters cannot distinguish prognostically favorable from prognostically unfavorable tumors. Since the expression of LSECtin ligands is associated with a significantly worse prognosis, the expression of LSECtin ligands could affect therapeutic decisions in the future.

CD301 binds the structures Tn, Sialyl-Tn, and 5NeuGcTn (van Vliet et al. 2005; Jegouzo et al. 2013; Mortezai et al. 2013). In the past, the Tn and STn structures were detected in carcinomas of the breast and in other carcinomas using antibodies of varying specificity. Depending on the specificity of the antibodies and the composition of the patient populations, contradictory results have been obtained. In a compilation of clinical results on the binding of STn antibodies to the corresponding ligands in breast carcinomas, the positivity rate ranged from 20% to 85% (Julien et al. 2012). It is not surprising that with this range of variation, statements on the clinical significance of the expression of the Tn and STn structures in breast carcinomas are contradictory. So far, no results are available for the clinical significance of the Neu5Gc-Tn structure in breast cancer.

For ligands of the CD301 glycan-binding receptor, we find a correlation between ligand expression and prognosis only in the patient group with triple negative breast cancer (TNBC). In the estrogen receptor-positive tumors, the expression of CD301 ligands had no prognostic significance. In contrast to our current results, we previously reported on a weak, positive correlation between CD301 positivity and prognosis in breast cancer (Kurze et al. 2019). This discrepancy may be explained by the small number of samples (*n* < 150) and broad spectrum of breast cancer subtypes investigated in this study, emphasizing the importance of analyzing large cohorts with well-defined subgroups of breast cancer patients as presented in this study.

In a clinical study by Leivonen et al., high expression of STn correlated with estrogen and progesterone hormone receptor negativity (Leivonen et al. 2001). Since TNBC tumors are associated with a worse prognosis overall than other breast carcinomas, our result is particularly relevant clinically. Festari et al. report on a higher tumorigenicity of a Tn-positive TNBC cell line compared to a Tn-negative TNBC cell line in a syngenic mouse model. Interestingly, Tn + tumor cells induce an immunoregulatory environment with higher expression of FoxP3 in CD4+ T cells, both in primary tumors and metastatic lungs (Festari et al. 2022).

In contrast to CD301, there is as yet no information on the clinical significance of the ligands of LSECtin in breast carcinomas. This may be due to the fact that so far there are no antibodies that specifically bind the LSECtin ligands. This is where the particular strength of our methodological approach becomes apparent. The probes of glycan-binding receptors allow statements to be made about the expression of biologically relevant glycans, even if there are no methodological alternatives for detecting the structures in question.

Recently, transcriptomes were analyzed on the single cell level in different types of breast cancer (Wu et al. 2021). Both, *CLEC4G* and *CLEC10A*, the genes coding for LSECtin and CD301, were expressed almost exclusively in the myeloid cell cluster. *CLEC4G* mRNA expression was most frequent in EGR1+ and LAM2_APOE+ macrophages as well as in cycling and SIGLEC+ cells. Gene expression was not significantly different between the different cancer subtypes. As expected, *CLEC10A* is a marker of cDC2 cells (Heger et al. 2018). However, in this cell population, the levels of *CLEC10A* expression did not differ significantly between the ER+ / HER2+ and TNBC subtypes. In contrast, the macrophage subtypes positive for LAM2_APOE, LAM1_FAB5 and EGR1, respectively, prevailed in TNBC. Interestingly, the LAM1_FAB5 signature correlated with worse survival (Wu et al. 2021). It is tempting to speculate that different subpopulations of *CLEC10A*-expressing cells may explain the prognostic differences between *CLEC10A* positive ER+ / HER2+ and TNBC tumors. However, future studies of breast cancer cell subtypes are required to proof the association between expression of glycoreceptor, their ligands, frequencies of immune cells and prognosis. Alternatively, the prognostic difference may be due the fact that effective targeted therapies are available for ER+ and HER2+ breast cancer.

The findings that increased, rather than decreased, expression of glycan-binding receptor ligands in subgroups of breast carcinomas is associated with poor prognosis is particularly relevant. These findings suggest that the ligands play an active role in cancer progression. The ligands of both LSECtin and CD301 were found to have an immunosuppressive function. For example, LSECtin inhibited the proliferation of tumor-specific effector T cells in melanomas, thereby promoting immune escape (Xu et al. 2014). Tn-bearing glycoproteins, such as MUC1 or CD43, interact with CD301 on macrophages to cause increased production of IL-10 and apoptosis of effector T cells (van Vliet et al. 2006; van Vliet et al. 2013). The increased expression of LSECtin and CD301 ligands could thus cause an inhibition of the immune response comparable to an immune checkpoint (RodrIguez et al. 2018). If, as supposed, the ligands of the glycan-binding receptors exert the function of an immune checkpoint, there would be a realistic therapeutic perspective here for inhibiting the checkpoint.

The methodological approach presented here can be applied to other glycan-binding receptors and other tumor entities. Based on large numbers of tumors with well-documented courses, reliable conclusions can be drawn on the diagnostic and prognostic significance of tumor-associated glycans. This will deepen our understanding of the biological significance of tumor-associated glycans in cancer and open up new avenues of tumor therapy.

## Material and methods

### Tissue and patient samples

In this study, paraffin-embedded tissue-microarrays (TMAs) of in total 1,859 formalin-fixed human breast cancer tissues were used. TMAs consisted of 50–100 tissue samples of 1 mm in diameter plus 4 positive control spots. Selection criteria of the cases, spot number and the number of events per TMA are presented in Table S2.

Specifically, the G1 gray zone encompasses well-differentiated (grade 1) carcinomas of no specific histological type (NST) with a tumor size ranging from 0.5 cm to 2 cm. This size range corresponds to stage pT1b and pT1c according to the current TNM classification of malignant tumors at the time of diagnosis, as defined by the International Union Against Cancer (IUCC) (Brierley 2016). Additionally, these carcinomas in the G1 gray zone do not exhibit lymph node metastases (pN0).

The G2 gray zone represents carcinomas of intermediate (grade 2) histological grade (NST) with a tumor size between 0,5 cm and 5 cm (pT1b, pT1c and pT2), also without histologically proofed lymph node metastases (pN0).

The G3 gray zone is composed of poorly differentiated (grade 3) tumors (NST) with a size between 2–5 cm (pT2) and at least one lymph node metastasis.

We also analyzed a cohort (*n* = 300), hereafter termed “endocrine only,” comprising pT1a—pT2 moderately differentiated (grade 2) tumors, independent of lymph node status, that received endocrine monotherapy.

In addition, we analyzed a “mixed cohort” (*n* = 284) compiled of unfiltered breast cancer samples of various histological subtypes irrespective of their grade, tumor size, and lymph node status, consisting of NST, mucinous, tubular, lobular, metaplastic, and invasive neuroendocrine carcinomas.

The tissue collection which was provided by MVZ Prof. Niendorf Pathologie Hamburg West GmbH included consent forms of all patients and follow up studies. The study was conducted according to the guidelines of the Declaration of Helsinki, and approved by the Ethics Committee of Ärztekammer Hamburg (PV 2946; date of approval: 2008 May 22 [initial application], 2010 July 13 [extension for prospective part], 2015 February 26 [update of study documents and continuation]).

### Glycan-binding receptor labeling, ELISA and immunohistochemical staining

The extracellular domains of human C-type glycoreceptors CD301 (Q8IUN9; aa 67–316), Langerin (Q9UJ71; aa 71–328), LSECtin (Q6UXB4; aa 59–293), DC-SIGNR (Q9H2X3; aa 274–390), and SRCL (Q8K4Q8; aa 63–742) were amplified by PCR from a human cDNA pool library (Clontech; Mountain View, CA), cloned into the eukaryotic EBB expression vector with N-terminal IgK-leader and myc-tag as previously described (Bogoevska et al. 2006; Samsen et al. 2010; Nollau et al. 2013). Constructs were transiently expressed in HEK293T cells, recombinant glycan-binding receptors were harvested from tissue culture supernatants and complexed prior to staining by a biotinylated anti-myc antibody (clone 9E10, sc-40-B, Santa Cruz, Dallas, Texas, USA) followed by incubation with HRP-conjugated streptavidin (Thermo Fisher Scientific, Waltham, Massachusetts, USA).

Glycan ELISA was performed as previously described (Nollau et al. 2013). In brief, 2 μg of recombinant proteins or 1 μg per well of poly(N-hydroxyethyl acrylamide) (PAA) conjugated glycoconjugates (Lectinity, Moscow, Russia), were coated on maxisorb 96-well plates (Thermo Fisher Scientific), blocked with carbo-free blocking solution (Newark, California, USA) and incubated with the preformed glycoreceptor complex in Tris saline magnesium (TSM)-buffer 20 mM Tris/HCl (pH 7.4), 150 mM NaCl supplemented with 0.1% Tween-20. Binding was analyzed in parallel in the presence of 2 mM MgCl_2_, 1 mM CaCl_2_ or 1% 0.5 M EDTA. For detection, ABTS solution (Roche, Mannheim, Germany) was added as substrate and absorbance was measured at 405 nm on a microplate reader. 10 μg biotinylated lectins PNA and GNL (Vector Laboratories, Newark, California, USA) were complexed with 1 μg Streptavidin-HRP (Thermo Fisher Scientific) in 100 μL TSM-buffer. Recombinant human CD44 (ACROBiosystems, Newark, Delaware, USA), Ebola virus glycoprotein 1 (GP1_EBOV; Sino Biological Europe, Eschborn, Germany), glucose oxidase (GOX_ASPNG) derived from Aspergillus niger, and L1CAM Fn 2–3 produced in HEK293S GnTI- (CVCL_A785) (Guedez et al. 2023) was used. Indicated glycoproteins were coated, blocked and digested using PNGase F (New England Biolabs, Frankfurt am Main, Germany) for 1 h at 37 °C.

For the detection of glycans in paraffin embedded tissue, protein domain histochemistry (PDH) was applied to 21 TMAs and 2 control TMAs as previously described (Nollau et al. 2013). In brief, sections (4 μm) were deparaffanized in 100% xylol and rehydrated through a graded ethanol series. For unmasking of epitopes, antigen retrieval was performed by boiling in 0.1 M citrate buffer (pH 6.0) for 40 min at 95 °C. Endogenous peroxidases were blocked by incubation in 3% hydrogen peroxide for 10 min at RT. Subsequently, sections were washed in TSM-buffer for 2x5min. To avoid unspecific binding, blocking was performed in carbo-free blocking solution for 1 h at room temperature. After removal of blocking solution, slides were incubated with 300 μL of the GBR-complex in TSM-buffer containing 2 mM MgCl_2_, 1 mM CaCl_2_ or 1% 0.5 M EDTA (pH 7.4) for 2 h at RT. To minimize variation in staining, we stained all TMAs simultaneously for each GBR including the EDTA controls. We labeled each GBR freshly and used only one preparation for the entire set of TMAs. After washing in TSM-buffer, DAB (3,3′-diaminobenzidine) was applied for detection of GBR binding. Finally, sections were counterstained with haemalaun, dehydrated and permanently covered. Chemicals were purchased from Carl Roth GmbH (Karlsruhe, Germany), Sigma-Aldrich (St. Louis, Missouri, USA) and Th. Geyer GmbH & Co. KG (Berlin, Germany).

### Manual and computer assisted evaluation

Immunohistochemical stainings and tissue microarrays (TMAs) were documented using the 3D HisTech Panoramic 1000 scanner at 40× magnification. The images were generated using the 3D HisTech software and CaseViewer Native Windows Application (Case Viewer Ink.). The evaluation of TMAs was done manually and with computer assistance.

For the computer-assisted evaluation, we employed the following process: The digitized TMAs in MIRAX file format were used as input for the digital image analysis. With the help of QuPath software (Bankhead et al. 2017), we extracted the individual cores of the patients as TIFF images using a semi-automatic method. A color separation algorithm, similar to the one described by Macenko et al. (Macenko et al. 2009), was applied to the RGB images. This algorithm identified the prominent stain colors and transformed the RGB color space into the color space of the stains. The algorithm produced two grayscale images, one representing the intensity of the positive stain color and the other representing the intensity of the negative stain color. In the case of glyco-stains, blue corresponded to stained nuclei, while brown/yellow represented positive-stained cells. The blue nuclei were segmented using the StarDist model (Schmidt et al. 2018), a convolutional neural network that imposed star-convex conditions on nucleus segmentation. A watershed algorithm was then applied to approximate the cell bodies in areas with high cell densities. Thereby, the cell outlines were positioned halfway between adjacent nuclei. With this approximation for the cell bodies, we analyzed the positive stain intensities within those regions. To decide whether a cell was positively or negatively stained, a robust criterion was used: the sum of all positive intensities within a cell needed to exceed the threshold of 4. This criterion accounted for different levels of staining intensity, such as 100% intensity for more than 0.25 μm^2^ of the cell, 50% intensity for 0.5 μm^2^ of the cell, and 25% intensity for 1 μm^2^ of the cell (see Fig. 2B). The number of positive and negative cells per patient was counted, and the ratio of these numbers (positive cells divided by negative cells) was used as a prognostic measure. To establish a universal threshold for each staining (CD301, LSECtin, Langerin), 50 equidistant thresholds were tested within the entire collective of cases. The threshold interval was defined to include at least 15% of patients in one risk group. The best threshold, determined by minimum log-rank p value, was then tested in each subcollective (e.g. grayzone or TNBC). The results were presented using a Kaplan-Meier plot. The relevant data is presented in Table S3.

### UMAP visualization

All available datasets from GEO accession number GSE176078 were downloaded and further analyzed in R environment v4.1.1 with the Seurat package v4.0.5. First, the single datasets were merged into one dataset without any batch correction and afterwards processed by selecting 2000 variable features, 1:50 dimensions and a resolution of 0.5 as parameters. To get the myeloid cell cluster, only cells with UMAP_1 > 1 and UMAP_2 > 5 were chosen, to remove few, presumably wrong clustered or annotated cells.

## Supplementary Material

Supplementary_Materials_R1_cwae003

Supplementary_Table_I_cwae003

Supplementary_Table_II_cwae003

Supplementary_Table_III_cwae003

Supplementary_Table_IV_cwae003

## Data Availability

The authors declare that all data supporting the findings of this study are available within the article, including its supplementary information files, or are available from the authors upon request.
