# Supplementary material for: CD301 and LSECtin glycan-binding receptors of innate immune cells serve as prognostic markers and potential predictors of immune response in breast cancer subtypes
Source: Glycobiology. 2024 Jan 11;34(3):cwae003. doi: 10.1093/glycob/cwae003 (PMC10987291; doi:10.1093/glycob/cwae003)
Supplement: Supplementary_Materials_R1_cwae003 [file supplementary_materials_r1_cwae003.pdf]

Supplementary Materials

CD301 and LSECtin glycan-binding receptors of innate immune cells serve as prognostic markers and potential predictors of immune response in breast cancer subtypes

Anne-Sophie Wegscheider, Irina Wojahn, Pablo Gottheil, Michael Spohn, Joseph Alfons Käs, Olga Rosin, Bernhard Ulm, Peter Nollau, Christoph Wagener, Axel Niendorf and Gerrit Wolters-Eisfeld

This file includes:

Figure S1: Assay validation and characterization of glycan-binding receptor complexes.

Figure S2: Establishment of the protein domain staining method using glycan-binding receptors as probes.

Figure S3: Background binding of SRCL in different tissues.

Figure S4: Langerin is no potential diagnostic marker in breast cancer subtypes.

Figure S5: *CLEC10A* and *CLEC4G* expression in the myeloid cluster.

Figure S6: Relative levels of *CLEC10A* and *CLEC4G* expression.

Figure S7: UMAP visualization of *CLEC4G* expression in the myeloid cell cluster.

Supplementary Table I: Composition and results of test-TMAs staining.

Supplementary Table II: Summary of the composition of breast cancer TMAs.

Supplementary Table III: Raw data of manual and IT-based evaluation including statistical analyses.

Supplementary Table IV: Summary *CLEC10A* and *CLEC4G* expression levels myeloid cluster.

Figure S1

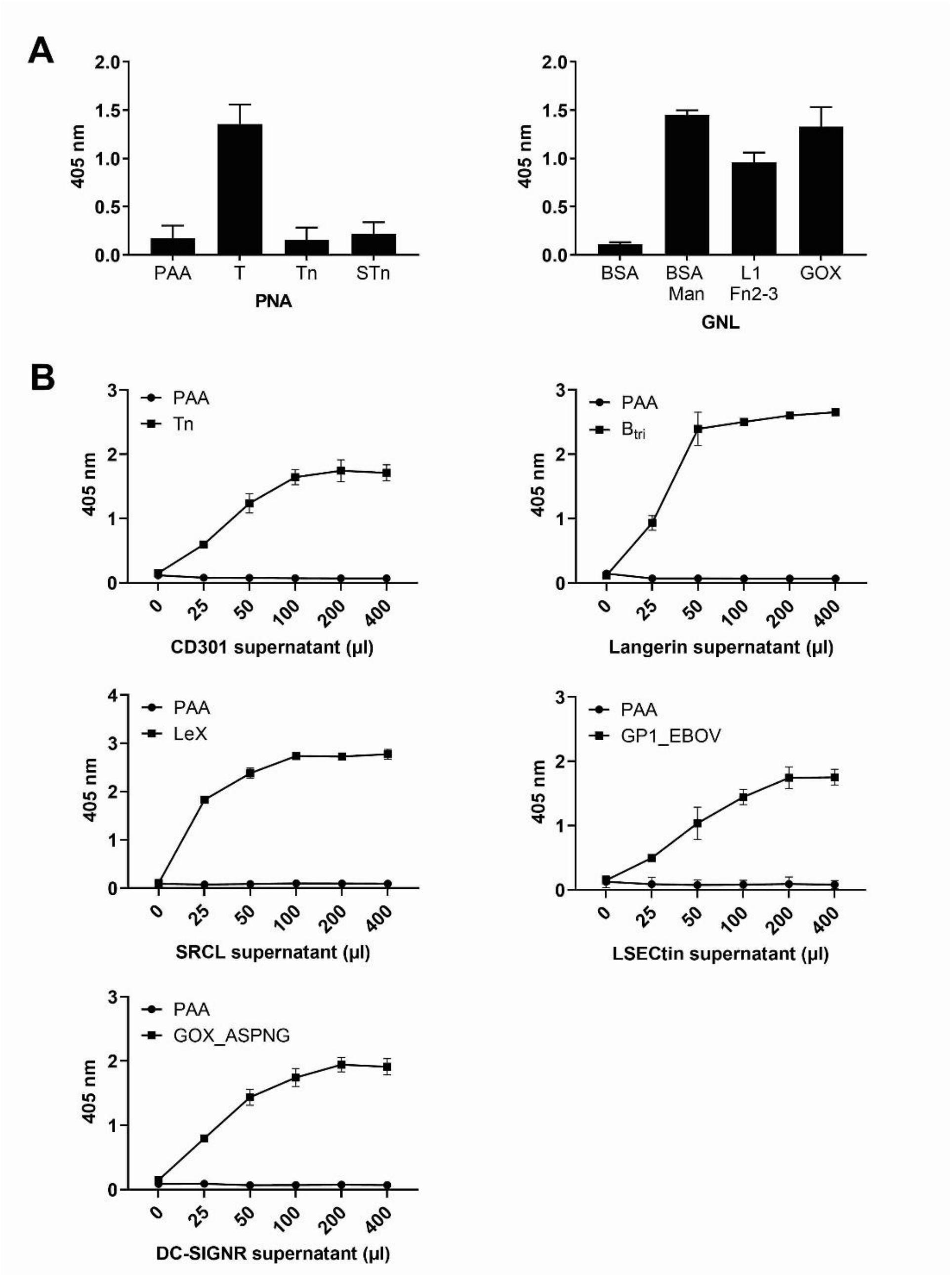

Fig. S1. Assay validation and characterization of glycan-binding receptor complexes.

(A) The negative control spots in the glycan ELISA were effectively probed using biotinylated plant lectins with well-defined glycan specificities. PNA specifically binds to the T antigen, while GNL exhibits binding affinity towards mannose and high mannose BSA conjugates. (B) Dilution curves of glycan-binding receptor complexes, which selectively bind to a defined glycan or glycoprotein rather than the control, illustrate asymptotic dose-response curves, thereby indicating equilibrium conditions.

Figure S2

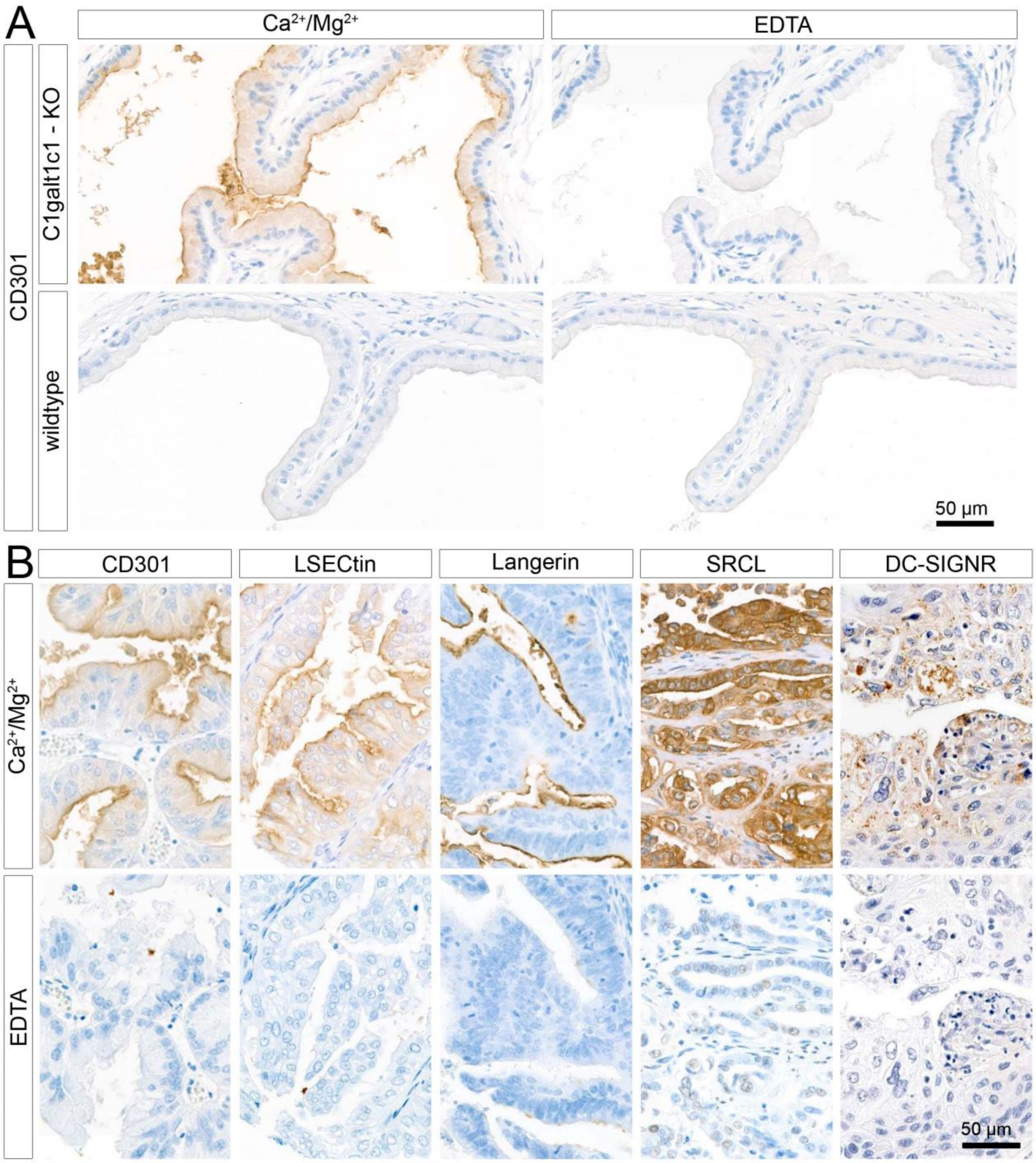

Fig. S2. Establishment of the protein domain staining method using glycan-binding receptors as probes.

(A) Establishment of CD301 binding in pancreatic tissues derived from conditional transgenic *C1galt1c1* knock out mice (positive control) and wild type (negative control) in the presence of Ca^{2+}/Mg^{2+} or EDTA. A positive signal was only observed in the tissue of *C1galt1C1*-KO mice overexpressing Tn antigen bound by CD301 in presence of divalent ions. (B) Detection

of positive signals on different human tissue samples using CD301 (here: ovarian carcinoma), LSECtin (here: ovarian carcinoma), Langerin (here: endometrium carcinoma) SRCL (here: endometrium carcinoma), DC-SIGNR (here: lung carcinoma) as probes.

Figure S3

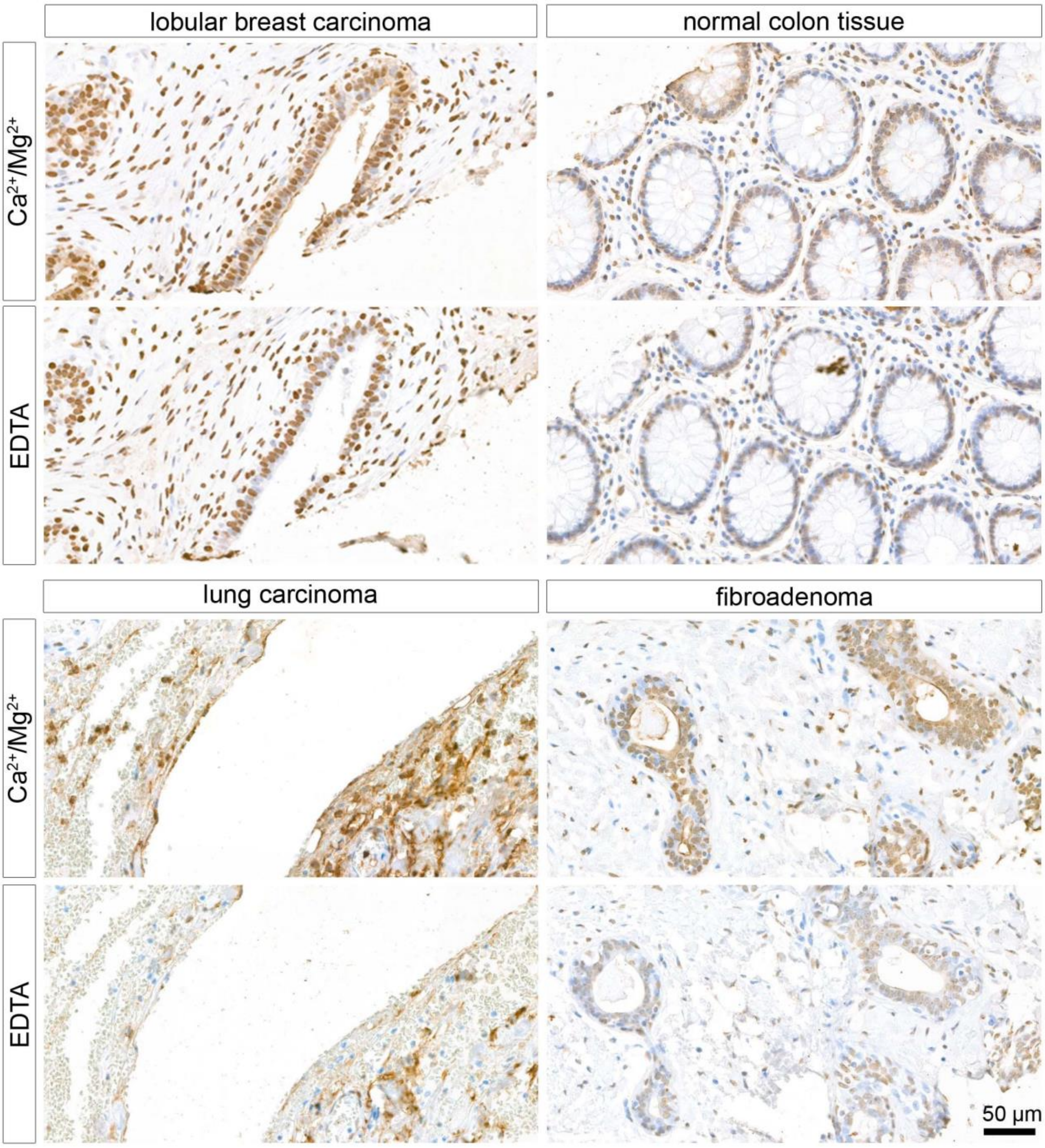

Fig. S3. Background binding of SRCL in different tissues.

This background staining was consistently observed in various tissues under both Ca^{2+}/Mg^{2+} and EDTA conditions. The signal appeared prominently in the nuclei as well as in other cellular compartments. As a result, the SRCL probe was deemed unsuitable for further analysis using algorithm-based methods. Tissue sample, buffer conditions and scale are as indicated in the figure.

Figure S4

A

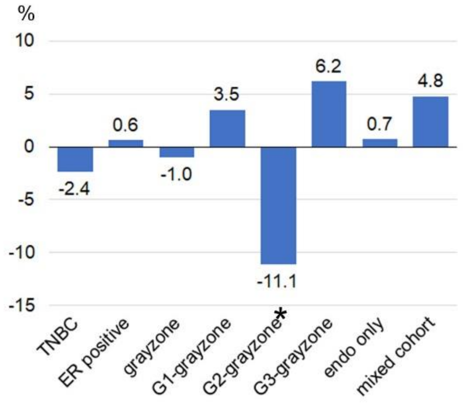

B

group	subgroup	p-value
TNBC		0.9040
ER$^+$		0.8503
	G1	0.5441
grayzone	G2	0.4468
	G3	0.2931
endocrine only		0.6123
mixed cohort		0.9273
**all cases**		**0.6258**

Fig. S4. Langerin is no potential prognostic marker in breast cancer subtypes.

(A) Frequencies of Langerin staining in different breast cancer subgroups in comparison to the average staining frequencies. (B) Summary of Kaplan-Meier log-rank p-values for the different breast cancer subgroups.

Figure S5

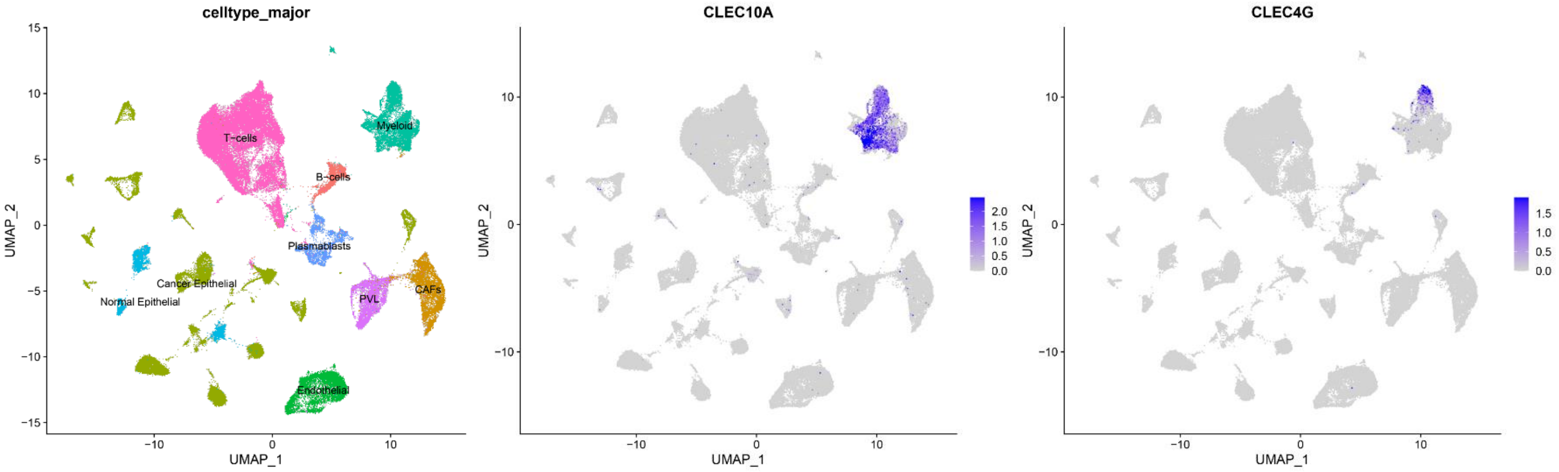

Fig. S5. CLEC10A and CLEC4G expression in the myeloid cluster in comparison with all other cell clusters assigned by single cell sequencing of TNBC and HER2+/ER+ breast cancer.

Figure S6

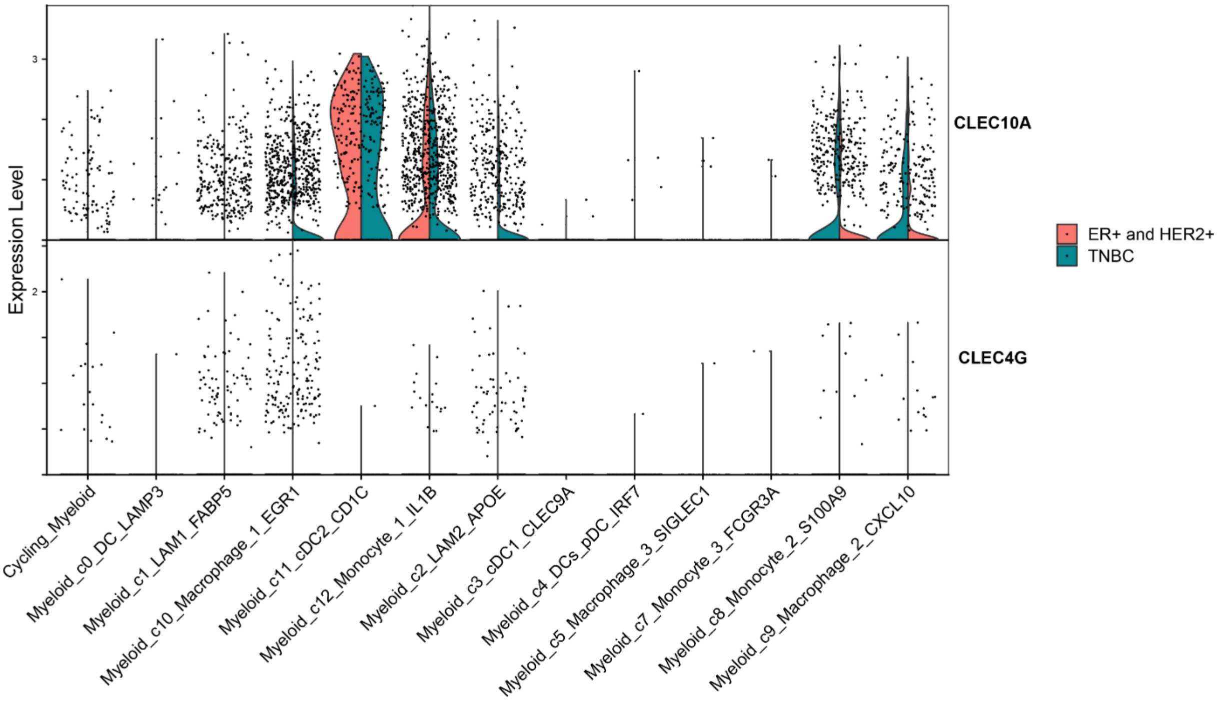

Fig. S6. Relative levels of CLEC10A and CLEC4G expression between HER2+/ER+ breast cancer cells and TNBC in different cell type cluster of the myeloid.

Figure S7

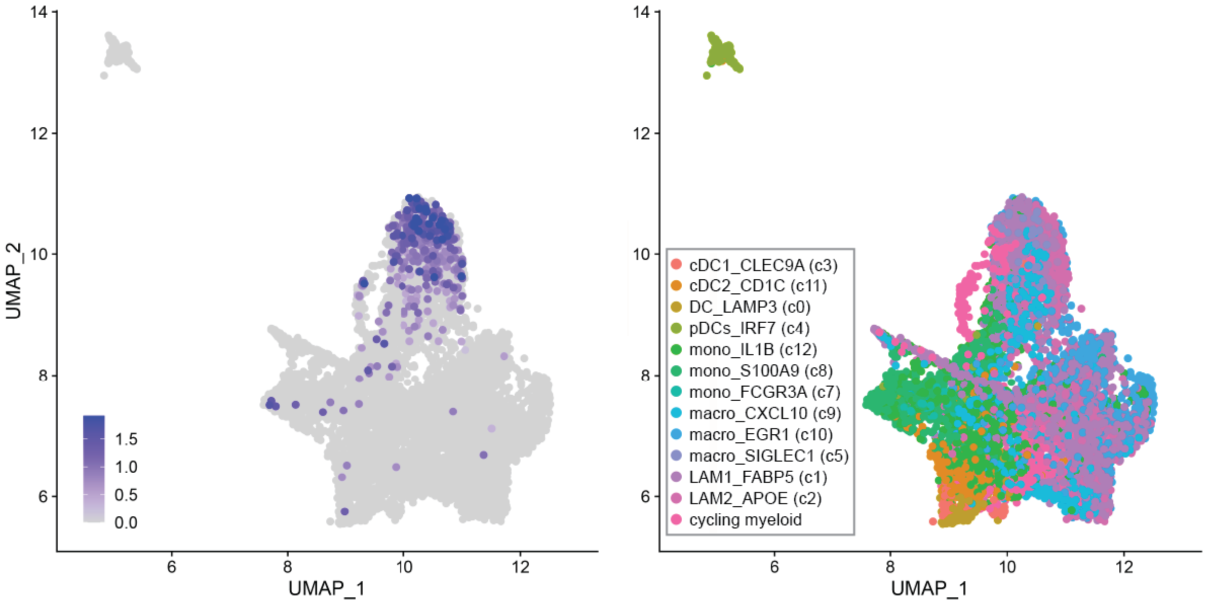

Fig. S7. UMAP visualization of CLEC4G expression in the myeloid cell cluster.
